# Taking Cold

**Published:** 1871-09

**Authors:** 


### TAKING COLD.

"The least thing in the world gives me a cold," is the complaint of many, especially of those who "house" themselves too much — who are on their feet but very little. Such persons should make it a point to walk out two hours every forenoon, and an hour in the afternoon, taking care to cool off slowly afterwards; and if, in addition, such persons were to dip the end of a towel in cold water, press out the water so as to prevent dripping, lay it on the head, and rub the whole surface of the body, as far as can be reached, in every direction, on rising and retiring, the pestersome habit of taking cold from every wind that blows would be broken up in a month in very many cases. The more a person bundles up, the more the slightest puff of air is avoided, the more easily will colds be taken, and before one knows it, the habit is so inveterate that the avoidance against taking cold becomes the main study of life. Such should fear the fate of Farquhar McKenzie, who more than thirty years ago took to his bed, and as a cold affected him in the most painful manner, he guarded against it more and more, until he found himself in a condition that his hands had to be kept gloved all the time; the curtains were always drawn around the bed, and the only access to him was by means of a pane of glass, inserted for that purpose. And yet he lived thus thirty years, dying a month ago, showing to what the human system can become habituated.

